# IUC24417-83 Correlation of circulating tumor DNA with oncological endpoints in metastatic urothelial cancer: a systematic review and meta-analysis of phase II/III clinical trials

**DOI:** 10.1093/oncolo/oyaf248.011

**Published:** 2025-08-29

**Authors:** S Mehrotra, A C Redla, J K Tan, A Ghose, A Maniam, S Boussios, G L Banna, P Das, S Adeleke, A Sharma

**Affiliations:** King’s College London, London, United Kingdom; King’s College London, London, United Kingdom; University of Manchester, Manchester, United Kingdom; Barts Cancer Centre, London, United Kingdom; Portsmouth Hospitals University NHS Trust, Portsmouth, United Kingdom; University of Ioannina, Ioannina, Greece; Portsmouth Hospitals University NHS Trust, Portsmouth, United Kingdom; University Hospitals of Derby, and Burton NHS Foundation Trust, Derby, United Kingdom; Cancer Centre at Guy’s, London, United Kingdom; Mount Vernon Cancer Centre, Northwood, United Kingdom

## Abstract

**Background:**

Metastatic urothelial cancer (mUC) bears a poor prognosis, with current surveillance strategies offering limited precision. Biomarkers such as circulating tumor DNA (ctDNA) are emerging tools to monitor treatment response in early disease, albeit their role in the advanced setting is under exploration. Our objective was to systematically review and correlate blood-derived ctDNA levels with primary and/or surrogate oncological endpoints in mUC.

**Methods:**

Our systematic review searched the MEDLINE database for phase II/III clinical trials comprising adult patients with mUC (population) undergoing ctDNA analysis (intervention) vs standard of care, that is, no ctDNA involvement (comparator). More than 1500 records were reviewed from 2006 to May 2025, with 16 being selected for full text screening and 2 meeting inclusion criteria. A meta-analysis using RStudio assessed the correlation between ctDNA levels and survival outcomes.

**Results:**

Two multi-center clinical trials—a dose-escalation and dose-expansion, phase I/II (Study 1108, NCT01693562, PMID 30093454) (*n* = 29) and a randomized controlled phase III (KEYNOTE-361 study, NCT02853305, PMID 38823511) (*n* = 260) were selected, resulting in 289 mUC patients undergoing ctDNA testing. CtDNA levels were measured as maximum variant allele frequency (MaxVAF).

In terms of overall survival (OS), meta-analysis showed that baseline low MaxVAF had a pooled hazard ratio (HR) of 0.84 (95% CI, 0.52-1.35; *P* = .47). This suggests a 16% reduction in the hazard of death vs high MaxVAF counterparts, although not statistically significant. Moderate heterogeneity (*I*^2^ = 61%) between trials reflected variability. In terms of progression-free survival (PFS), meta-analysis demonstrated that baseline low MaxVAF had a pooled HR of 1.24 (95% CI, 0.64-2.41; *P* = .52), implying a 24% increase in the hazard of disease progression or death vs the high MaxVAF group. There was no statistical significance. High heterogeneity (*I*^2^ = 79%, *P* = .029) limited interpretability.

**Conclusions:**

Lower baseline ctDNA levels (MaxVAF) inversely correlated with better OS outcomes without statistical significance, whereas association with PFS was inconclusive and/or showed nil consistent effect. Current evidence is insufficient to support ctDNA as a reliable prognostic biomarker in the advanced urothelial cancer setting, warranting further investigation.

